# An Alternative Pathway for Formononetin Biosynthesis in *Pueraria lobata*

**DOI:** 10.3389/fpls.2016.00861

**Published:** 2016-06-14

**Authors:** Jia Li, Changfu Li, Junbo Gou, Xin Wang, Rongyan Fan, Yansheng Zhang

**Affiliations:** ^1^CAS Key Laboratory of Plant Germplasm Enhancement and Specialty Agriculture, Wuhan Botanical Garden, Chinese Academy of Sciences, HubeiChina; ^2^University of Chinese Academy of Sciences, BeijingChina

**Keywords:** hairy root, isoflavone, 4′-*O*-methylation, *O*-methyltransferase, *Pueraria lobata*

## Abstract

The *O*-methylation is an important tailing process in *Pueraria lobata* isoflavone metabolism, but the molecular mechanism governing it remains not elucidated. This manuscript describes the mining of key *O*-methyltransferases (OMTs) involved in the process. Using our previously constructed *P. lobata* transcriptome, the OMT candidates were searched, extensively analyzed, and their functions were investigated by expression in yeast, *Escherichia coli*, or *Glycine max* hairy roots. Here, we report the identification of the key OMT gene responsible for formononetin production in *P. lobata* (designated as *PlOMT9*). PlOMT9 primarily functions as an isoflavone-specific 4′-*O*-methyltransferase, although it shows high sequence identities with isoflavone 7-*O*-methyltransferases. Moreover, unlike the previously reported OMTs that catalyze the 4′-*O*-methylation for formononetin biosynthesis at the isoflavanone stage, PlOMT9 performs this modifying step at the isoflavone level, using daidzein rather than 2,7,4′-trihydroxy-isoflavanone as the substrate. Gene expression analyses and metabolite profiling supported its proposed roles in *P. lobata*. Using the system of transgenic *G. max* hairy roots, the role of PlOMT9 in the biosynthesis of formononetin was further demonstrated *in vivo*.

## Introduction

**FIGURE 1:**
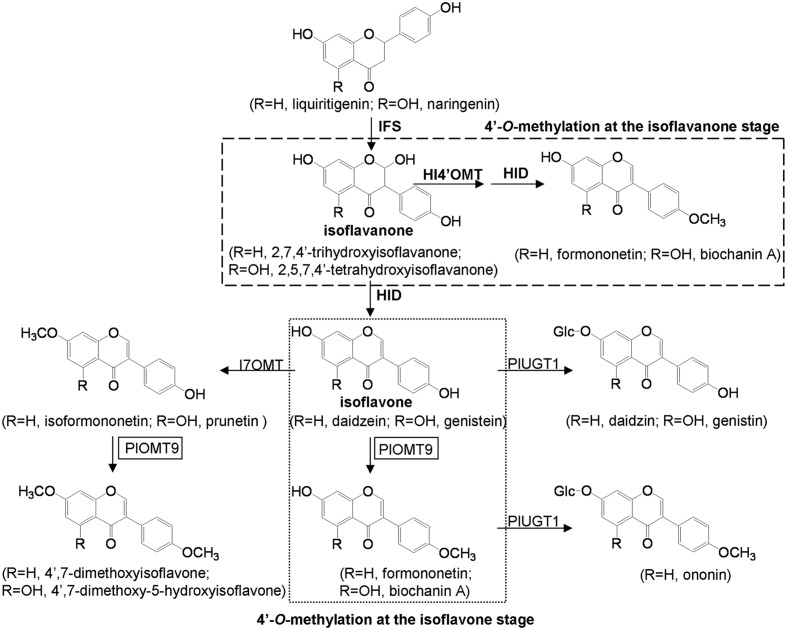
**Putative pathways of isoflavonoid biosynthesis in *P. lobata*.** IFS, isoflavone synthase; HI4′OMT, 2,7,4′-trihydroxy-isoflavanone 4′-*O-*methyltransferase; I7OMT, isoflavone 7-*O*-methyltransferase; HID, tri-hydroxy-isoflavanone dehydratase; PlUGT1 (Li J. et al., 2014), isoflavone 7- *O*-glucosyltransferase; PlOMT9, isoflavone 4′-*O*-methyltransferase (this study).

Isoflavonoids are specialized metabolites primarily produced in leguminous plants (Veitch, 2007). Many of them have a multitude of possible functions in plant ecological adaptation (Phillips and Kapulnik, 1995) and human-health-promoting activities (Lamartiniere, 2000; Merz-Demlow et al., 2000). Starting from common flavanone intermediates (liquiritigenin or naringenin), the backbone of isoflavonoids is formed by a cytochrome P_450_ isoflavone synthase (IFS), yielding 2-hydroxy-isoflavanone that is not chemically stable and readily dehydrated to form isoflavones (daidzein or genistein). The isoflavone scaffold is further decorated by glycosyltransferases and methyltransferases, contributing to the chemical diversification of isoflavonoids (**Figure 1**). *O*-methylation is mediated by *O*-methyltransferases (OMTs). Most of the OMTs bear strict substrate specificities and define specific physiological roles *in vivo* (Ibrahim et al., 1998; Bureau et al., 2007). For example, pea 6α-hydroxy-maackiain 3-OMT catalyzes the final *O*-methylation step in the biosynthesis of the antimicrobial compound pisatin, suggesting a role for OMTs against biotic stresses in plants (Wu et al., 1997). Methylated isoflavonoids also show medicinal effects such as anti-cancer (e.g., formononetin), neuroprotective (e.g., biochanin A), antifungal (e.g., 4′,7-dimethoxyisoflavone), and osteogenic (e.g., isoformononetin) activities (Pandey et al., 2002; Kumar et al., 2011; Lo and Wang, 2013; Tan et al., 2013).

*Pueraria lobata* (*P. lobata*) is a leguminous plant that produces large quantities of glycosylated isoflavonoids with important pharmacological activities (Lin et al., 1996; Keung and Vallee, 1998; Jin et al., 2012). It also produces several types of isoflavonoids that are methylated at their C-7, 4′, and 3′ positions. The 4′-*O*-methylated isoflavones (e.g., formononetin and biochanin A) are main methylated isoflavonoids in *P. lobata*, while other *O*-methylated isoflavonoids, such as 7-*O*- (e.g., isoformononetin), 4′, 7-di-*O*- (e.g., 4′,7-dimethoxyisoflavone), and 3′-*O*-methylated isoflavones (e.g., 3′-methoxy-daidzein, 3′-methoxy-daidzin and 3′-methoxy-puerarin), were also reported from this plant but in less amounts (Rong et al., 1998; Dubey et al., 2008). Isoflavonoid 7-*O*-methyltransferases (I7OMTs) and 2,7,4′-trihydroxy-isoflavanone 4′-*O*-methyltransferases (HI4′OMTs) have received some attention in several leguminous plant species, including *Medicago sativa*, *Glycyrrhiza echinata*, *Lotus japonicus*, and *Medicago truncatula* (He et al., 1998; Akashi et al., 2003; Deavours et al., 2006). For the I7OMT, its cDNA from alfalfa (*M. sativa*) has been cloned and the gene product methylates the 7-hydroxyl group of daidzein to form isoformononetin *in vitro* (He et al., 1998). For the HI4′OMTs, experiments with *G. echinata*, *L. japonicus*, and *M. truncatula* demonstrated that both natural and recombinant HI4′OMTs recognized 2,7,4′-trihydroxy-isoflavanone but not daidzein (an isoflavone) as the direct methyl acceptor, strongly suggesting that HI4′OMTs performs the 4′-*O*-methylation for the biosynthesis of formononetin at the isoflavanone stage (**Figure 1**; Akashi et al., 2003; Deavours et al., 2006).

With the goal of understanding the methylation of isoflavonoids in *P. lobata* at molecular levels, we recently have constructed *P. lobata* transcriptomes by RNA sequencing (Wang et al., 2015), which allowed us to identify the genes involved in the methylation process in *P. lobata*. Here, we report the successful identification of a key gene responsible for formononetin biosynthesis in *P. lobata* (designated *PlOMT9*). PlOMT9 exhibits high amino acid identities with I7OMTs, but it primarily functions as an isoflavone 4′-*O*-methyltransferase (I4′OMT) while only retains trace of I7OMT activity. More interestingly, not like the previously identified HI4′OMTs (Akashi et al., 2003; Deavours et al., 2006), PlOMT9 performs the 4′-*O*-methylation for formononetin biosynthesis at the isoflavone level, using daidzein rather than 2,7,4′-trihydroxy-isoflavanone as the substrate. Transgenic experiments with *Glycine max* hairy roots also demonstrated a role of *PlOMT9* in formononetin biosynthesis *in vivo*.

## Materials and Methods

### Plant Materials and Chemical Sources

*P. lobata* seeds were collected from Anhui Province of China and grown in soil under normal controlled conditions as described previously (Li J. et al., 2014). *G. max* seeds were provided by Prof. Jian Zhao at the Huazhong Agricultural University of China. The chemical standards prunetin and biochanin A were purchased from BioBioPha Co. Ltd (Kunming, China), 4′,7-dimethoxy-5-hydroxyisoflavone were from Tianjin Sigma Technology Co. Ltd (Tianjin, China), 4′,7-dimethoxyisoflavone was from Tianjin Yifang Technology Co. Ltd (Tianjin, China) and all the other chemical standards were purchased from Shanghai Source Leaf Biological Technology Company (Shanghai, China). All organic solvents used for high-performance liquid chromatography (HPLC) and liquid chromatography–mass spectrometry (LC–MS) were of HPLC grade and they were purchased from Wuhan Dingguo Biotechnology Co. Ltd (Wuhan, China). All the restriction endonucleases were from Takara Company (Takara, Dalian, China). All the nucleotides used for the PCRs of this study are shown in Supplementary Table S1.

### Molecular Cloning and Phylogenetic Analysis of OMT Candidates

A transcriptome derived from the roots and leaves of *P. lobata* was constructed by our group and its raw RNA sequences have been deposited at the sequence read archive (SRA) of the National Center for Biotechnology Information (accession number: SRX480408). The coding region sequences of *PlOMTs* were discovered from the transcriptome, amplified by RT-PCRs from the *P. lobata* roots, and cloned into the pMD18-T vector (Takara, Dalian, China) for a sequencing confirmation. The thermocycling conditions for the PCRs were used as follows: at 95°C for 3 min for one cycle; at 95°C for 15 s, then at 55°C for 30 s and at 72°C for 2 min for 35 cycles, and a final elongation step at 72°C for 10 min.

For the phylogenetic analysis, the deduced amino acid sequences of the OMT candidates from *P. lobata* were aligned with other plant OMTs with known functions using the CLUSTAL X Version 2.0 program (Larkin et al., 2007). The phylogenetic tree was constructed by the neighbor-joining method of the MEGA 6.0 program (Tamura et al., 2013). The information of the sequences used in the phylogenetic analysis is summarized in Supplementary Table S2.

### Functional Analysis of OMT Candidates in Yeast

For the expression in yeast cells, the coding regions of *PlOMTs* were amplified and sub-cloned into the yeast expression vector pESC-HIS (Stratagene, La Jolla, CA, USA) to give the constructs pESC-HIS-PlOMTs. The plasmids pESC-HIS-PlOMTs and empty vector pESC-HIS were introduced separately into the yeast WAT11 strain (Pompon et al., 1996). Yeast cultures were grown in His dropout liquid medium (Clontech, Palo, CA, USA) containing 2% (w/v) glucose. After 24 h in a shaker maintained at 250 rpm, the yeast cells were collected by centrifugation, washed with sterile water for three times, and re-suspended to an OD_600_ of 0.8 in 5 ml of His dropout liquid medium containing 2% (w/v) galactose supplemented with the substrates such as daidzein, genistein, formononetin, isoformononetin, prunetin, and biochanin A, separately, at a final concentration of 100 μM. After incubation at 30°C for 48 h, the yeast cultures were extracted with an equal volume of ethyl acetate, the acetate extracts were air dried at room temperature and re-dissolved in methanol for HPLC analysis.

### Expression and Purification of Recombinant OMTs

The coding regions of *PlOMT2*, *PlOMT9*, and *HI4′OMT* (Deavours et al., 2006) were amplified, and sub-cloned into the *Escherichia coli* expression vector pET28a in frame with an N-terminal His_6_ tag, yielding the plasmids pET28a-PlOMT2, pET28a-PlOMT9, and pET28a-HI4′OMT. These constructs were then transformed into *E*. *coli* BL21 (DE3) cells using heat shock at 42°C and the expression of the recombinant OMTs was induced with 0.5 mM IPTG at 16°C for 14 h. Cells were centrifuged at 6,000 rpm (4,629 × *g*) at 4°C for 10 min. The resulting cell pellets were re-suspended in 20 ml of lysis buffer consisting of 20 mM sodium phosphate (pH 7.4), 300 mM sodium chloride, and 10 mM imidazole. Cells were lyzed with lysozyme on ice for 30 min followed by sonication on ice by 1-s pulse for 30 min. The cell-free extracts containing recombinant OMTs were obtained by centrifugations at 4°C for 30 min. The recombinant OMTs were then purified using HisPur^TM^ Ni-NTA Resin (Thermo, Waltham, MA, USA) following the provided protocol. The purity of the recombinant OMTs was checked by SDS-PAGE and their concentrations were determined by Bradford assays using BSA as the protein standard. For the peptide analysis of recombinant PlOMT2, its corresponding stained band was excised from the SDS-PAGE gel, digested with trypsin, and analyzed by MALDI-TOF/TOF mass spectrometry according to the method previously described (Li et al., 2012).

### Enzyme Assays *In Vitro*

Unless otherwise stated, the *in vitro* enzyme assays were carried out in a 200 μl reaction mixture consisting of 0.5–5μg of the purified OMT, 50 mM Tris–HCl (pH 8.0), 2 mM DTT, 2 mM S-adenosyl methionine (SAM), and 100 μM substrates. The reactions were performed at 37°C for 20 min and stopped by adding 200 μl of methanol. The reaction time of 20 min was determined to be in the linear range for the activities by preliminary experiments. The 15 μl of the reaction mixture was then directly used for HPLC and LC—MS analysis.

For the assays with 2,7,4′-trihydroxy-isoflavanone, the reaction mixture consisted of 200 μg of the yeast microsomes expressing *P. lobata* IFS (GenBank accession number KC202929) prepared as previously described (Liu et al., 2003), 5 μg of the purified recombinant OMTs, 50 mM Tris–HCl (pH 8.0), 2 mM NADPH, 2 mM DTT, 2 mM SAM, and 100 μM liquiritigenin. The reaction was allowed to proceed at 30°C for 60 min, stopped by 200 μl of methanol, and was directly applied for HPLC and LC–MS analysis.

### Measurement of Metabolite Concentration and Gene Expression in *P. lobata*

Plant materials (roots, stems, and leaves) of *P. lobata* grown at open field were collected, powdered under liquid nitrogen, and stored at –80°C for the measurement of methylated isoflavone formononetin and the *PlOMTs* gene expression. The increment of isoflavonoid biosynthesis in *P. lobata* in response to methyl jasmonate (MeJA) elicitation was previously reported (Thiem and Krawczyk, 2010; Li Z.B. et al., 2014). For the MeJA treatment in this study, a stock solution of MeJA was first dissolved in ethanol, and added into liquid 1/2 MS medium where one-month-old *P. lobata* seedlings were grown. The final concentration of MeJA in the MS medium was 100 μM and the concentration of ethanol in the medium was 0.001% (v/v). For the mock controls, the same concentration of 0.001% ethanol was added into the liquid MS medium. Roots were collected after 6 h treatment for the gene expression analysis, and after 10-day treatment for the measurement of methylated isoflavones. For gene expression analysis, total RNA was isolated and treated with DNase I. Transcript levels were analyzed by quantitative RT-PCR (q RT-PCR). For the measurement of methylated isoflavones, 20 mg of plant materials was extracted with 1 ml of ethanol with three biological repeats. The ethanol extracts were air dried at room temperature and re-dissolved in methanol for HPLC and LC–MS analysis. Two-tailed *t* test (confidence interval: 95%) was performed using GraphPad Prism 5 software for the statistical analysis of the data.

### Generation of Transgenic *G. max* Hairy Roots

The coding region of *PlOMT9* was PCR amplified and fused with the open reading frame of green fluorescent protein (GFP) in the plant expression vector pCAMBIA1302 (CAMBIA, Canberra, Australia) by enzyme digestions and ligations, yielding the plasmid pCAMBIA1302-PlOMT9. The *Agrobacterium rhizogenes* strain K599 was separately transformed with pCAMBIA1302-PlOMT9 as well as the empty vector pCAMBIA1302 as a control.

The hairy roots of *G. max* were generated using these transgenic *A. rhizogenes* strains, following previously described methods (Cho et al., 2000). After around 3–4 weeks from the inoculation, hairy roots appeared on infection sites. When these roots were at least 2 cm long, the positive transgenic hairy roots were identified by genomic PCRs. A segment from each positive root was excised and sub-cultured in 1/2 MS liquid medium with antibiotics in the dark. After 2 weeks of the sub-culture, those transgenic hairy root cultures were treated with MeJA at a final concentration of 100 μM. As a mock control, the same volume of ethanol was added into the cultures. The transgenic roots were used for metabolite analysis after 10 days of the treatments. For the metabolite analysis, 36 positive roots per each construct were cultured with 6 roots per 150 ml flask in a shaker, leaving that the half of the root cultures (18 roots in three flasks as 3 biological replicates) was used for the MeJA-mediated elicitation and the other half of the cultures was treated as the mock controls. Hairy roots from each flask were collected, powdered together, and extracted with ethanol for the quantification of relevant metabolites, which includes daidzein, daidzin, formononetin, and ononin.

### Quantitative RT-PCR

Total RNAs were isolated from roots, stems, and leaves of *P. lobata* using Trizol (Invitrogen) and treated with DNase I (Thermo) to remove contaminated genomic DNA. First-strand cDNA was prepared using superscript III reverse-transcriptase (Invitrogen). The *P. lobata* actin gene (GenBank accession number HO708075) was used as an internal standard to normalize the variation in each cDNA preparation. The qRT-PCR was performed on an ABI 7500 Fast Real-Time PCR Detection System with FastStart Universal SYBR Green Master mix (Rox; Roche, Mannheim, Germany). The PCR conditions were as follows: 10 min of initial denaturation at 95°C, followed by 40 cycles of 95°C for 15 s and then 60°C for 1 min. All the RT-PCRs were performed in three biological repeats. Two-tailed *t* test (confidence interval: 95%) was performed using GraphPad Prism 5 software for the statistical analysis of the data. Gene-specific primers were used for the qRT-PCRs and are listed in Supplementary Table S1.

### HPLC and LC–MS/MS Analysis

HPLC analysis was performed on an LC-20AT instrument (Shimadzu, Kyoto, Japan) using an inertsil ODS-SP reverse-phase column (250 mm × 4.6 mm, 5 μm). The column temperature was set at 30°C. Samples from the *in vitro* enzyme assays were eluted in the mobile phase, 0.4% phosphoric acid (solvent A) and HPLC-grade methanol (solvent B), with 65% B for 45 min for the reactions with genistein and 60% B for 40 min for the other reactions (with quercetin, kaempferol, and luteolin) at a flow rate of 0.8 ml min^-1^. For other analyses, 0.1% (v/v) formic acid (A) and acetonitrile (B) were used as the mobile phase and samples were separated at a flow rate of 0.8 ml min^-1^ in a stepped gradient mode as follows: 0–30 min, 25–90% B; 30–35 min, 90–25% B; 35–40 min, 25% B; and the flow rate was 0.8 ml min^-1^. The detection wavelength was set at 260 nm for daidzein, genistein, prunetin, formononetin, isoformononetin, and biochanin A; at 280 nm for liquiritigenin; at 350 nm for apigenin and luteolin; at 370 nm for quercetin, kaempferol, and isoliquiritigenin. A standard curve was acquired from different concentrations (5–100 μg ml^-1^) of each chemical standard for the quantification.

LC–MS/MS analysis was performed on an Accela LC system coupled with TSQ Quantum Access Max mass spectrometer (Thermo Scientific, USA) and electrospray ionization source. The column and the analysis method were the same as the HPLC analysis described above. The MS data were recorded in a positive ion mode with ranges of *m/z* 50–500.

## Results

### Identification of (Iso)flavonoid OMT Candidates in the *P. lobata* Transcriptome

**FIGURE 2:**
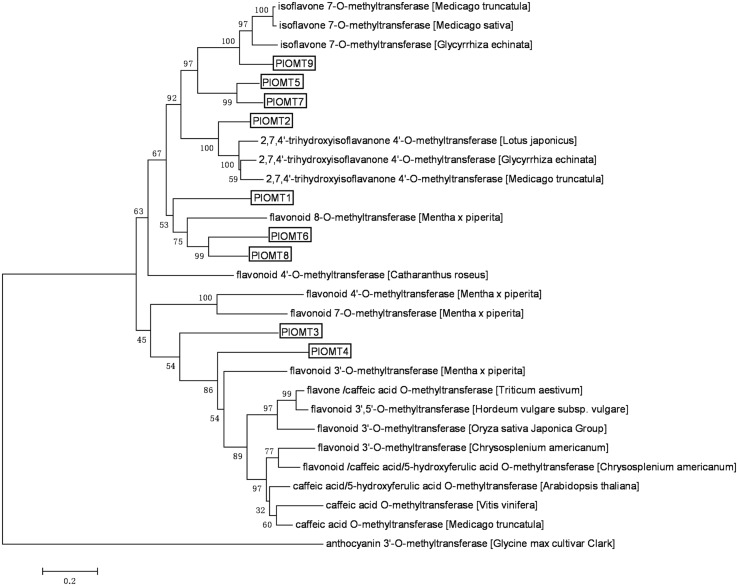
**Phylogenetic analysis of the PlOMTs with other OMTs whose functions are already known.** Amino acids were aligned using the CLUSTAL X Version 2.0 program, and the phylogenetic tree was constructed by the neighbor-joining method of MEGA 6.0. Numbers on each node indicate the bootstrap values of 1000 replicates. The scale bar represents 0.2 amino acid substitutions per site.

Analysis of the transcriptome database derived from the roots and leaves of *P. lobata* (GenBank accession number: SRX480408; Wang et al., 2015) revealed the presence of 108 unigenes annotated as OMTs (Supplementary Table S3), of which only 42 OMT unigenes are in a length of more than 1 kb. We decided to select the OMT candidates from these 42 unigenes. The criteria to select the OMT candidates are as follows: (i) the target genes would be transcribed at relatively higher levels in *P. lobata* roots than its leaves, given the fact that the isoflavonoids of *P. lobata* mainly accumulate in its roots (Kirakosyan et al., 2003; Matkowski et al., 2003); (ii) the OMT candidates would be annotated as enzymes related to (iso)flavonoid metabolisms by BLAST searches against public databases. Finally, nine OMT candidates were identified. Full-length cDNAs for these nine OMTs (termed PlOMT1-9, GenBank accession numbers, KP057884–KP057892) were presented in the dataset and their deduced amino acids share 21–81% identities with each other. The relationship of the OMTs candidates with other plant OMTs was assessed through phylogenetic analysis of deduced polypeptide sequences (**Figure 2**). PlOMT1, PlOMT6, and PlOMT8 clustered with flavonoid 8-*O*- and 4′-*O*-methyltransferases. PlOMT2 occurred in the same branch with HI4′OMTs whose functions are known to be responsible for the 4′-methylation in formononetin biosynthesis at the isoflavanone stage (Akashi et al., 2003; Deavours et al., 2006). PlOMT5, PlOMT7, and PlOMT9 could be grouped into the clade with I7OMTs. Enzymes in this subclass are believed to mediate the 7-*O*-methylation of isoflavones, such as daidzein (He et al., 1998; Akashi et al., 2003; Deavours et al., 2006). PlOMT3 and PlOMT4 showed a closer relationship to flavonoid 3′-*O*-methyltransferases, which indicates possible roles for PlOMT3 and PlOMT4 in 3′-*O*-methylation of (iso)flavonoids. As a focus on the investigation on the 4′- and/or 7-*O*-methylation processes here, all the PlOMTs described above, except for PlOMT3 and PlOMT4, were investigated in this study.

### Biochemical Characterization of the OMT Candidates

**FIGURE 3:**
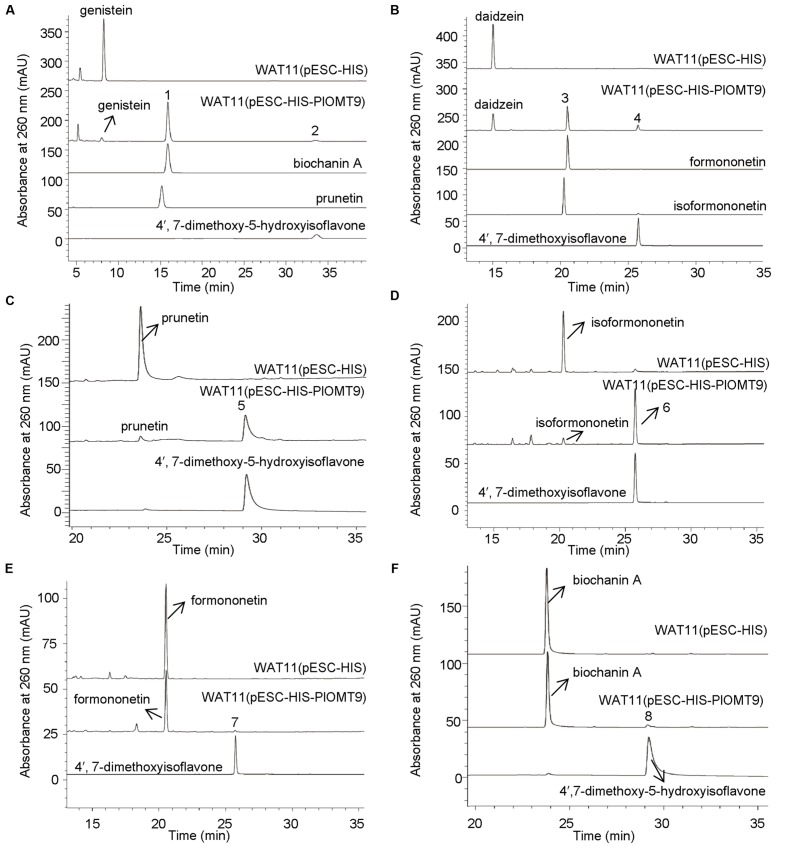
**LC-MS analysis of the products from the *in vivo* assays in yeast cells. (A)–(F)** show the products of the PlOMT9-expressed cells fed with the substrates genistein yielding biochanin A (peak 1) and 4′,7-dimethoxy-5-hydroxyisoflavone (peak 2), daidzein yielding formononetin (peak 3) and 4′,7-dimethoxyisoflavone (peak 4), prunetin yielding 4′,7-dimethoxy-5-hydroxyisoflavone (peak 5), isoformononetin yielding 4′,7-dimethoxyisoflavone (peak 6), formononetin yielding 4′,7-dimethoxyisoflavone (peak 7), and biochanin A yielding 4′,7-dimethoxy-5-hydroxyisoflavone (peak 8), respectively. WAT11 (pESC-HIS) represents the strain carrying the empty vector pESC-HIS as a control; WAT11 (pESC-HIS-PlOMT9) represents the strain expressing PlOMT9. The mass spectra of the peaks 1–8 and their corresponding authentic standards are shown in Supplementary Figure S1.

**FIGURE 4:**
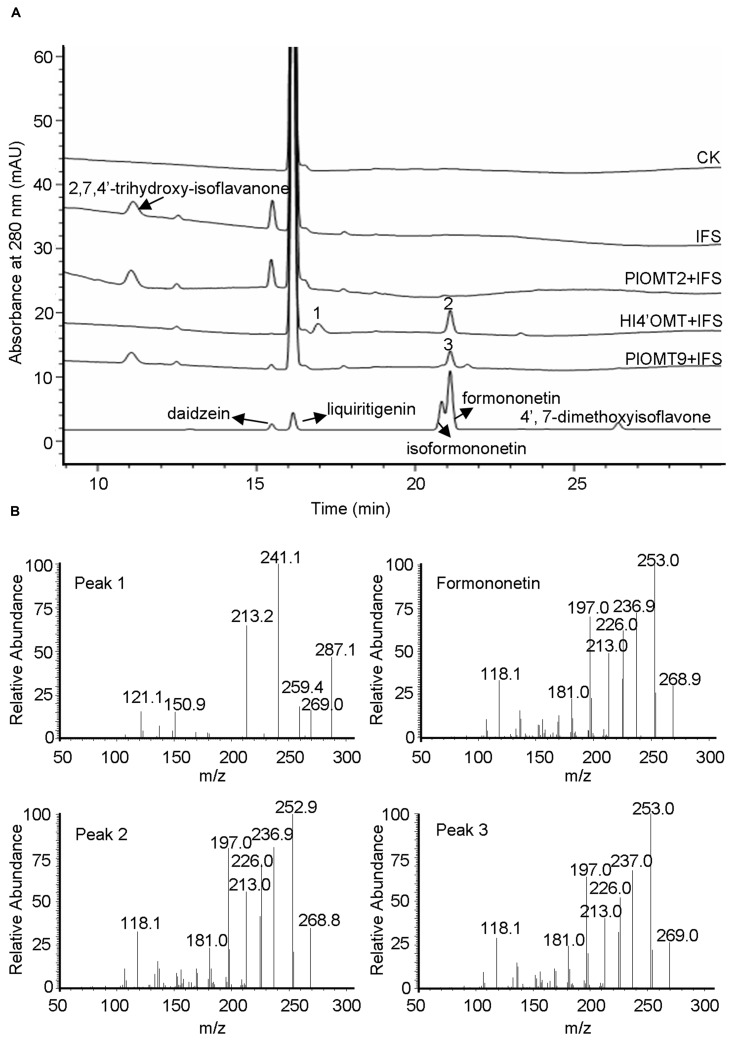
**LC-MS analysis of the products from the *in vitro* assays of the purified recombinant PlOMT2, PlOMT9 or HI4′OMT with 2,7,4′-trihydroxy-isoflavanone. (A)** HPLC profiles were shown for the production of 2,7-dihydroxy-4′-methoxy-isoflavanone (peak 1) and formononetin (peak 2) in the reaction with HI4′OMT, formononetin (peak 3) in the reaction with PlOMT9, and no enzymatic products in the reactions with PlOMT2; **(B)** the mass spectra of peaks 1–3 and formononetin standard; The substrate 2,7,4′-trihydroxy-isoflavanone was prepared by the incubation of the yeast microsome expressing *P. lobata* IFS (GenBank accession number KC202929) with a racemic mixture of *2R/S*-liquiritigenin and NADPH; The collision energy in the LC-MS analysis is 15 V for 2,7-dihydroxy-4′-methoxy-isoflavanone and 30 V for formononetin.

To screen the biochemical activities of the OMT candidates, the PlOMT cDNAs were inserted into the vector pESC-HIS (Stratagene, La Jolla, CA, USA) for expression in *Saccharomyces cerevisiae* WAT11 (Pompon et al., 1996), the yeast transformed with the empty vector pESC-HIS served as the control. The activities of PlOMTs were tested by adding various substrates (daidzein, genistein, formononetin, isoformononetin, prutein, or biochanin A) to yeast cultures that express recombinant PlOMTs, followed by testing the conversion of the substrates using HPLC analysis. Notably, yeast cells expressing PlOMT9 was able to metabolize part of the substrates (**Figure 3**), and no measurable activities were observed for the other PlOMTs (data not shown). Given the closer relationship of PlOMT9 to I7OMTs in the phylogenetic tree (**Figure 2**), PlOMT9 was initially presumed as an I7OMT. Unexpectedly, the *in vivo* assays showed that yeast expressing PlOMT9 efficiently methylated the 4′-hydroxy groups of genistein, daidzein, prunetin, and isoformononetin, forming their respective 4′-*O*-methylated isoflavones biochanin A (peak 1), formononetin (peak 3), 4′,7-dimethoxy-5-hydroxyisoflavone (peak 5), and 4,7-dimethoxyisoflavone (peak 6), respectively (**Figures 3A–D**). These data indicated that PlOMT9 primarily functions as an I4′OMT but not as an I7OMT as expected. Interestingly, when genistein or daidzein was fed, the cells bearing PlOMT9 also produced trace amounts of 4′, 7-di-*O*-methylated products (peak 2 and peak 4; **Figures 3A,B**), suggesting that PlOMT9 retains trace of I7OMT activity. To clarify this, the substrates whose 4′-OH is already methylated, formononetin (4′-methoxy-daidzein) and biochanin A (4′-methoxy-genistein), were fed to the PlOMT9-expressed cultures, tiny peaks representing the products 4′, 7-dimethoxyisoflavone (7-*O*-methylated formononetin, (peak 7; **Figure 3E**) and 4′,7-dimethyoxy-5-hydroxyisoflavone (7-*O*-methylated biochanin A, peak 8; (**Figure 3F**) were observed. Therefore, our data clearly suggested that PlOMT9 primarily functions as an I4′OMT but retains the trace I7OMT activity. The identities of all the catalytic products were confirmed by comparisons with their respective authentic standards using LC–MS analysis (Supplementary Figure S1).

To analyze the substrate specificity of PlOMT9, the PlOMT9 fused with an N-terminal His_6_ tag was purified from an expression in *E. coli* cells by Ni-affinity chromatography (Supplementary Figure S2), and assayed with a broad range of substrates whose structures are shown in Supplementary Figure S3. As shown in **Table 1**, using SAM as the methyl donor, the purified PlOMT9 recognized 4′-hydroxy isoflavones as substrates and was found to be inactive with other types of compounds. Of the 4′-hydroxy isoflavones, the best substrate for PlOMT9 is genistein (100% relative activity, 7670.6 pkat mg^-1^) followed by daidzein (48.8% relative activity, 3742.9 pkat mg^-1^). Other isoflavones with a C-4′ hydroxyl group (prunetin and isoformononetin) are also utilized by PlOMT9 as acceptors but are poor substrates (5.2–6.2% relative activity; **Table 1**). When daidzein was used as the substrate for the *in vitro* assay, formononetin was predominantly formed while isoformononetin and 4′,7-dimethoxyisoflavone were also produced (Supplementary Figure S4). Again, this result further confirmed that PlOMT9 mainly functions as an I4′OMT with a trace I7OMT activity. The identities of all the catalytic products from the *in vitro* assays were confirmed by comparisons with their respective authentic standards using LC–MS analysis (Supplementary Figure S5).

**Table 1:** Enzyme activities of the recombinant PlOMT9 *in vitro.*

Substrate	Enzyme activity (pkat/mg)	Relative activity (%)^a^
***Isoflavone***		
Genistein	7670.6 ± 705.7	100 ± 9.2
Daidzein	3742.9 ± 145.7	48.8 ± 1.9
Prunetin	474.1 ± 38.2	6.2 ± 0.5
Isoformononetin	400.2 ± 38.4	5.2 ± 0.5
Biochanin A	0	0
Formononetin	0	0
***Flavone***		
Apigenin	0	0
Luteolin	0	0
***Flavanone***		
Liquiritigenin	0	0
***Flavonol***		
Kaempferol	0	0
Quercetin	0	0
***Chalcone***		
Isoliquiritigenin	0	0
***Phenolic acid***		
Caffeic acid	0	0

*^a^Relative activity. The values are normalized with the most converted substrate genistein as 100%.*

The intermediate 2,7,4′-trihydroxy-isoflavanone was previously proposed as a natural methylation acceptor for HI4′OMT from licorice (*G. echinata*; GenBank accession number BAC58011) and *M. truncatula* (GenBank accession number AAY18581). The 4′-methylation activity with 2,7,4′-trihydroxy-isoflavanone can be detected by the formation of the intermediate 2,7-dihydroxy-4′-methoxy-isoflavanone from *in vitro* enzyme assays (Akashi et al., 2003; Deavours et al., 2006). To examine the activity of PlOMT9 with 2,7,4′-trihydroxy-isoflavanone, 2,7,4′-trihydroxyisoflavanone was prepared by the incubation of the yeast microsome-expressing *P. lobata* IFS (GenBank accession number KC202929; Gou et al., 2013) incubated with a racemic mixture of *2R/S*-liquiritigenin and NADPH (Akashi et al., 1999). The previously characterized *M. truncatula* HI4′OMT (Deavours et al., 2006) was expressed and purified from *E. coli* cells as a positive control. In consistent with the previous report (Deavours et al., 2006), the product 2,7-dihydroxy-4′-methoxy-isoflavanone (peak 1) was clearly observed in the incubation with HI4′OMT and part of 2,7-dihydroxy-4′-methoxy-isoflavanone had dehydrated to formononetin (peak 2; **Figure 4**). In contrast, in the reactions with PlOMT9, the intermediate 2,7-dihydroxy-4′-methoxy-isoflavanone was not detected at all (**Figure 4**), suggesting that PlOMT9 is not able to methylate the substrate 2,7,4′-trihydroxy-isoflavanone. Formononetin (peak 3) was formed in the reaction with PlOMT9 (**Figure 4**), which probably resulted from the activity of PlOMT9 with daidzein that was formed by the spontaneous dehydration of 2,7,4′-trihydroxy-isoflavanone. In addition, considering the closer relationship of PlOMT2 to HI4′OMTs in the phylogenetic tree (**Figure 2**), we also prepared a purified recombinant PlOMT2 by expression in *E. coli* cells (Supplementary Figure S2) and assessed its activity with 2,7,4′-trihydroxy-isoflavanone, no enzymatic products by PlOMT2 were observed as well (**Figure 4**).

### The Relevance of PlOMT9 Transcripts to its Catalyzed Metabolites in *P. lobata*

**FIGURE 5:**
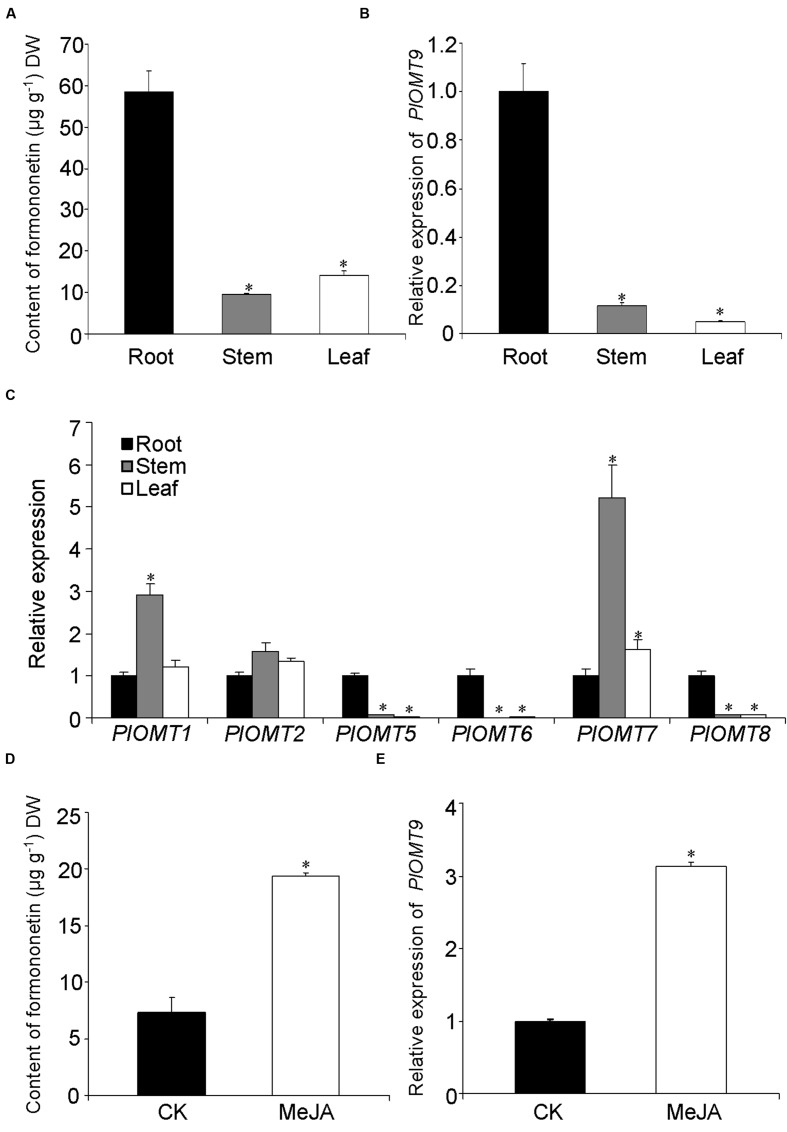
**Measurement of methylated isoflavone accumulation and gene expression in *P. lobata.* (A)** The concentration of formononetin in *P. lobata* organs analyzed by HPLC; **(B)** and **(C)**, the relative transcript abundance of the *PlOMTs* in *P. lobata* organs measured by qRT-PCRs; **(D)**, the effect of the MeJA treatment on the biosynthesis of formononetin in the *P. lobata* roots; **(E)**, the regulation of the MeJA elicitation on the gene expression of *PlOMT9* in the roots. The *P. lobata* actin gene (GenBank accession number HO708075) was used as an internal standard in the qRT-PCRs. Each measurement was performed in three biological replicates. Asterisks indicate significant differences, *P* < 0.05.

To examine the relevance of PlOMT9 to its methylated isoflavone biosynthesis in *P. lobata*, we measured the distribution of its *in vitro* enzymatic metabolite in various organs. As shown in **Figure 5A**, the formononetin (PlOMT9 product) was mostly detected in the root (58.4 ± 5.1 μg g^-1^ dry weight), but was almost undetectable in its aerial organs (stem and leaf). Expression analysis by quantitative reverse transcription-PCRs revealed that *PlOMT9* gene is specifically expressed in the root with extremely low levels in the stem and leaf (**Figure 5B**). Thus, in a spatial manner, the relative abundance of *PlOMT9* transcripts matches the accumulation pattern of formononetin in *P. lobata*, supporting the proposed roles of PlOMT9 physiologically. The gene expression pattern of all the other *PlOMT*s was also investigated. As shown in **Figure 5C**, *PlOMT5*, *PlOMT6*, and *PlOMT8* genes show preferential expression in the roots; the genes coding for *PlOMT1* and *PlOMT7* are transcribed at a higher level in the stem relative to the root and leaf while the *PlOMT2* gene shows similar expressions in all the three organs.

To further test the correlation in an eliciting manner, one-month-old seedlings of *P. lobata* were treated with 100 μM MeJA and their roots were then harvested for metabolite and nucleic acid extraction. The roots of 10-day-treated plants were used for metabolite extraction. The concentration of formononetin in the MeJA-treated plants apparently increased in comparison with the controls (**Figure 5D**). To examine the gene expressions, the roots of 6-h-treated plants were collected for this purpose. Relative to the control plants, the transcript level of *PlOMT9* was significantly elevated in response to the elicitation (**Figure 5E**). Taken together, the transcript abudance of *PlOMT9* correlates well with the accumulation pattern of formononetin in *P. lobata*. Two-tailed *t* test was performed and a *P* value of 0.05 was considered to be statistically significant.

### PlOMT9 Plays a Role for the Formononetin Biosynthesis in Planta

**FIGURE 6:**
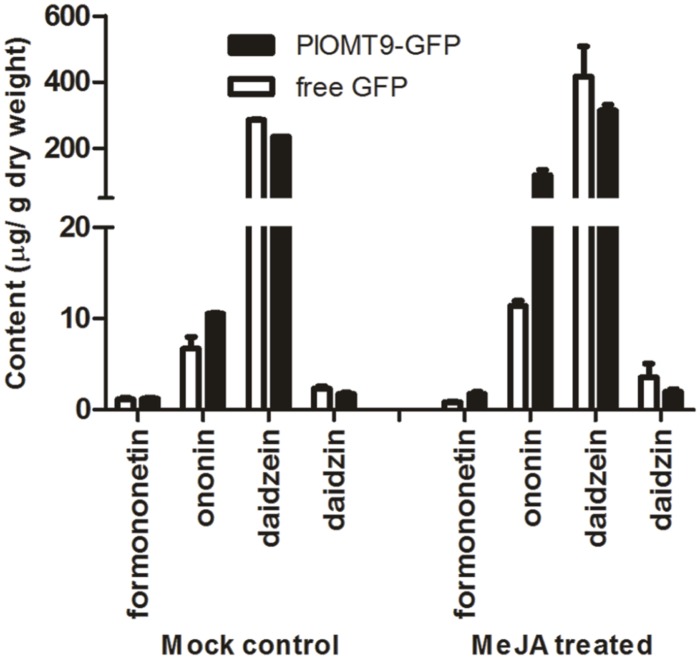
**The concentrations of formononetin, ononin, daidzein, and daidzin in transgenic soybean hairy roots.** PlOMT-GFP, the hairy roots transformed with pCAMBIA1302-PlOMT9; free GFP, the hairy roots transformed with the empty vector pCAMBIA1302; MeJA treatment, the hairy root cultures were treated with MeJA at the final concentration of 100 μM; Mock control, the hairy root cultures were treated with the same concentration of 0.001% ethanol. After 10 days of the treatments, the hairy roots were collected for measuring the metabolites by HPLC analysis. Each measurement was performed in three biological replicates.

To assess whether *PlOMT9* play a role for the formononetin biosynthesis *in planta*, *P. lobata* hairy roots were initially considered as the expression system for this purpose. However, our endeavor to establish *P. lobata* hairy roots failed although Liu et al. (2000) previously reported the success of *P. lobata* hairy root inductions. Upstream isoflavonoid biosynthesis pathway is conserved in legume species, and one of the legume species, *G. max*, also contains IFSs and its hairy roots physiologically provide daidzein (the *in vitro* substrate of PlOMT9 described above). Thus, the *G. max* hairy root was chosen as the expression host here as its hairy root system has been well established (Cho et al., 2000). The coding region of *PlOMT9* was fused to GFP in the vector pCAMBIA1302 and transformed into the hairy roots of *G. max*. The free GFP alone in the pCAMBIA1302 was expressed in hairy roots as the control. The presence of the transgenes was confirmed by genomic PCRs, and positive hairy roots were sub-cultured and treated with or without MeJA signal for metabolite quantification by LC–MS analysis. In the roots untreated with MeJA, the content of daidzein and daidzin was reduced by 17.5 and 26%, respectively, in the PlOMT9-expressed lines with respect to the control (**Figure 6**). On the other hand, compared to that of the control lines, the average concentration of formononetin in the PlOMT9-expressed roots was slightly increased (PlOMT9 expressed-lines, 1.20 μg g^-1^ DW; the control lines, 1.12 μg g^-1^ DW), whereas its 7-*O*-glucoside, namely ononin (**Figure 1**), was increased by 57.7% (PlOMT9-expressed-lines, 10.53 μg g^-1^ DW; the control lines, 6.68 μg g^-1^ DW; **Figure 6**). When the roots were elicited with MeJA, the roots expressing PlOMT9 produced significantly increased levels of both formononetin and ononin (formononetin and ononin were increased by 111.2 and 940.9%, respectively) compared to the control roots. Moreover, this increment was accompanied with the decrease in the biosynthesis of daidzein and daidzin (daidzein and daidzin were decreased by 24.3 and 45.2%, respectively; **Figure 6**). Taken together, all these data corroborated that PlOMT9 is involved in the biosynthesis of formononetin in planta.

## Discussion

*P. lobata* synthesizes 4′-*O*-methylated isoflavones such as formononetin and biochanin A (Rong et al., 1998; Dubey et al., 2008). In the legume species *G. echinata* and *M. truncatula*, the 4′-*O*-methylation appears to take place at the isoflavanone stage, using 2,7,4′-trihydroxy-isoflavanone rather than daidzein as the methyl acceptor (Akashi et al., 2003; Deavours et al., 2006). Genes encoding the HI4′OMT have been cloned from these two species (Akashi et al., 2003; Deavours et al., 2006). However, by a BLAST search, we did not find any HI4′OMT homologies in the EST databases (taxids: 3878, 3879, and 56066) of another legume species *M. sativa* that also produces 4′-*O*-methylated isoflavones (Liu and Dixon, 2001). To date, no HI4′OMT cDNAs have been isolated from *M. sativa*. This observation probably suggests that, not in all the legume species, the 4′-*O*-methylation is formed by HI4′OMTs. BLASTP search revealed that there was one HI4′OMT homology (73% amino acid identity), designated PlOMT2, presented in our constructed *P. lobata* cDNA database (accession number: SRX480408). However, biochemical assays showed that PlOMT2 was not active with 2,7,4′-trihydroxy-isoflavanone (**Figure 4**). Moreover, its expression is not in accordance with the accumulation of 4′-*O*-methylated isoflavones in different organs of *P. lobata* (**Figure 5**). These results suggested that PlOMT2 may not function as a HI4′OMT. On the other hand, unexpectedly, although the candidate PlOMT9 shows a high sequence identity with members of I7OMTs (**Figure 2**), it mainly functions as an I4′OMT, methylating daidzein to form formononetin (**Figure 3**). In addition, 2,7,4′-trihydroxy-isoflavanone, the substrate of HI4′OMT, could not be accepted by PlOMT9 (**Figure 4**). Taken together, these findings suggested that there is a possibility of isoflavones of some legume species being 4′-*O*-methylated by I4′OMTs at the isoflavone stage, while not necessarily by HI4′OMTs at the isoflavanone stage. The identification of PlOMT9 may indicate that, in some legume species, I7OMTs evolve to be I4′OMTs for the biosynthesis of 4′-*O*-methylated isoflavones during the evolutions.

The proposed 4′*-O*-methylation activity of PlOMT9 toward isoflavones was further supported by several observations from the *in vivo*. First, the PlOMT9 transcript abundance perfectly matches with the accumulation pattern of its enzymatic products in *P. lobata* (**Figure 5**), suggesting its role physiologically. The functional analysis of PlOMT9 was then extended by expressing it in soybean hairy roots. Compared with the control roots bearing the empty vector, the roots expressing PlOMT9 accumulated significantly higher levels of formononetin and its 7-*O*-glucoside ononin upon MeJA treatment. Moreover, this increment was accompanied with the decrease of the levels of daidzein and its 7-*O*-glucoside daidzin (**Figure 6**), further supporting the proposed role of PlOMT9 in plant. However, the role of PlOMT9 in formononetin biosynthesis still needs to be further proved by silencing its expression in *P. lobata*. In either *P. lobata* plant or the transgenic soybean hairy roots, the production of formononetin was elevated by MeJA treatment (**Figures 5D** and **6**). It is probable that *P. lobata* limits the methylation at normal conditions while only improves it to fight against stresses, as methylated isoflavones are considered as phytoalexins (Ibrahim et al., 1998; Bureau et al., 2007).

## Conclusion

We reported an alternative pathway for formononetin biosynthesis in nature in this study, which is different from that in *G. echinata* and *M. truncatula*. The alternative pathway was resulted from the 4′-*O*-methylation activity, which takes place at the isoflavone stage rather than at the isoflavanone stage, e.g., the PlOMT9 activity of *P. lobata* in this case.

## Author Contributions

YZ designed the project; JL performed the gene isolations and enzyme assays; CL carried out the gene expression analysis, *G. max* hairy root transformations; JG cloned the IFS gene; XW and RF provided assistance in preparing protein purification; YZ and JL wrote the manuscript.

## Conflict of Interest Statement

The authors declare that the research was conducted in the absence of any commercial or financial relationships that could be construed as a potential conflict of interest.
