# Supplementary material for: An Alternative Pathway for Formononetin Biosynthesis in Pueraria lobata
Source: Front Plant Sci. 2016 Jun 14;7:861. doi: 10.3389/fpls.2016.00861 (PMC4905983; doi:10.3389/fpls.2016.00861)
Supplement: Supplementary file 1 [file Data_Sheet_1.ZIP › Supplementary Material/Supplementary Material.doc]

Supplementary Material

**An alternative pathway for formononetin biosynthesis in *Pueraria lobata***

Jia Li^1a^, Changfu Li^1a^, Junbo Gou^1, 2^, Xin Wang^1^, Rongyan Fan^1, 2^, Yansheng Zhang^1^*

^a^ These authors contributed equally to this work.

***Correspondence:** Yansheng Zhang: [zhangys@wbgcas.cn](mailto:zhangys@wbgcas.cn)

# 1 Supplementary Figures and Tables

## 1.1 Supplementary Figures

**Supplementary Figure 1.** Mass spectrum of the enzymatic products in the yeast cultures expressing PlOMT9 fed with different substrates. (A), (C), (G) and (I), showed the mass spectra of the authentic biochanin A, 4′, 7-dimethoxy-5-hydroxyisoflavone, formononetin and 4′,7-dimethoxyisoflavone, respectively; Mass spectrums were shown for the products from the PlOMT9-expressed culture fed with the substrate genistein yielding biochanin A (peak 1) (B) and 4’,7-dimethoxy-5-hydroxyisoflavone (peak 2) (D), the substrate prunetin yielding 4’,7-dimethoxy-5-hydroxyisoflavone (peak 5) (E), the substrate biochanin A yielding 4’,7-dimethoxy-5-hydroxyisoflavone (peak 8) (F), the substrate daidzein yielding formononetin (peak 3) (H) and 4′, 7-dimethoxyisoflavone (peak 4) (J), the substrate isoformononetin yielding 4′, 7-dimethoxyisoflavone (peak 6) (K), the substrate formononetin yielding 4′, 7-dimethoxyisoflavone (peak 7) (L). The collision energy in the LC-MS/MS analysis is 30 V for the biochanin A, 4′, 7-dimethoxy-5-hydroxyisoflavone, formononetin and 4′, 7-dimethoxyisoflavone. The MS/MS data of the each product was consistent with corresponding authentic standard.

**Supplementary Figure 2.** SDS-PAGE electrophoresis of the purified recombinant proteins of PlOMT9 and PlOMT2*.* For the purification of the recombinant PlOMT2 from the *E. coli* cells, there are two major bands observed in the SDS-PAGE gel as shown in the figure, the two bands were excised from the gel, digested with trypsin, and analyzed by MALDI-TOF/TOF mass spectrometry. The peptides from the upper band matched an *E. coli* protein bifunctional polymyxin resistance protein arnA. The peptide sequences HVYNFMSSMALK, CAAELGIADAIHSHGKPMTLSELASALK, LLTHNGFFTK, GLGSLVDVGGGTGAVAR, FINEAFPHLK, VIIIDIVIDEAGDDR, and LIYDAGFSSYK, from the lower band matched the predicted amino acid sequences of PlOMT2, which suggested that the lower band corresponded to the recombinant purified PlOMT2.

**Supplementary Figure 3.** Structures of all the substrates used in the enzyme assays *in vitro*.

**Supplementary Figure 4.** *In vitro* enzyme assays of the purified recombinant PlOMT9 toward the substrate daidzein (A). (B) is the partially magnified part of (A). PlOMT9 converts daidzein to isoformononetin (peak 1), formononetin (peak 2) and 4′,7-dimethoxyisoflavone (peak 3). The mass spectra of the peaks 1-3 and their corresponding authentic standards were shown in Supplementary Figure 5.

**Supplementary Figure 5.** Mass spectra of the products shown in supplementary Figure 4**.** (A), (C) and (E) showed the mass spectra of the authentic isoformononetin, formononetin and 4′, 7-dimethoxyisoflavone, respectively; (B), (D) and (F) showed the mass spectra of the enzymatic products (peaks 1-3). The collision energy used for LC-MS/MS analysis is 30 V.

## 1.2 Supplementary Tables

**Supplementary Table 1. Primers used in this study**

| Genes | Primers 5' to 3' sequence |
| --- | --- |
| For pMD18-T /pESC-His |  |
| *PlOMT1* (upstream) | CCCGGATCCATGGAAAAGATGGATGCTGAAG |
| (downstream) | CCCGTCGACTCAAGGATAAACTTCAATAAGCGAC |
| *PlOMT2* (upstream) | CCCGGATCCATGGATTTCAGTAGCAATGGCA |
| (downstream) | CCCGTCGACTTAAGGATAAACTTCTATGAGAGATTTG |
| *PlOMT5* (upstream) | CCCATCGATATGGCTTCAATGAATAACCAA |
| (downstream) | CCCGAGCTCTTAAGAATAGACCTCAATCAGAGAT |
| *PlOMT6* (upstream) | CCCGGATCCATGGAATCCCATAGTGAAGA |
| (downstream) | CCCGTCGACCTATGGATAAACCTCGATGA |
| *PlOMT7* (upstream) | CCCGGATCCATGGCTTCAATGAATAACCAAG |
| (downstream) | CCCGTCGACCTAAGGATAGACCTCAATAAGAGAT |
| *PlOMT8* (upstream) | CCCGGATCCATGGAGCCCCAAAGTGAAGAG |
| (downstream) | CCCGTCGACTCAATGAGACCTTAAACCCAGCA |
| *PlOMT9* (upstream) | CCCGGATCCATGGGTTCAAATAATGGC |
| (downstream) | CCCGTCGACCTAAGGATAGATCTCAATAAGAG |
| For pET28a |  |
| *PlOMT2* (upstream) | CCCGGATCCATGGATTTCAGTAGCAATGGCA |
| (downstream) | CCCGTCGACTTAAGGATAAACTTCTATGAGAGATTTG |
| *PlOMT9* (upstream) | CCCGGATCCATGGGTTCAAATAATGGC |
| (downstream) | CCCGTCGACCTAAGGATAGATCTCAATAAGAG |
| *HI′4OMT* (upstream) | TCAGGATCCATGGCTTTCAGTACCAACGG |
| (downstream) | CCCGTCGACTCAAGGATAAACTTCGATGAGT |
| For pCAMBIA1302 |  |
| *PlOMT9* (upstream) | CCCGGATCCGATGGGTTCAAATAATGGC |
| (downstream) | CCCACTAGTAGGATAGATCTCAATAAGAGACA |
| For gene expression analyses |  |
| *Actin* (upstream) | GGGGTGATGGTTGGGATG |
| (downstream) | CGGTGAGAAGAACAGGGT |
| *PlOMT1* (upstream) | ATCACACCCTTTGACACTGC |
| (downstream) | TACACCTCCTTTACCTCCCC |
| *PlOMT2* (upstream) | GGGAAAGGGAAAGAAGGAAA |
| (downstream) | TGAAGCCAAATACAGGGGAA |
| *PlOMT5* (upstream) | CTCCAATGGTTCTAGGGTGT |
| (downstream) | TTTTGAGTGCCAAGTCTGTC |
| *PlOMT6* (upstream) | CAAGGAGGCAATAAAGAGCA |
| (downstream) | ACTGAAACCAGCAGAGAAAA |
| *PlOMT7* (upstream) | TGGTTTGGTTGAACACTGAT |
| (downstream) | CACTTGCCATACCCTCACTA |
| *PlOMT8* (upstream) | CAAGGAGGCAATAAAGAGCA |
| (downstream) | ACTGAAACCAGCAGAGAAAA |
| *PlOMT9* (upstream) | GTTGAGTGTATGCTTGACCCA |
| (downstream) | TTGAGAATCACTAGCCATTGC |

**Supplementary Table 2.** The methyltransferases used in the phylogenetic analysis

| GenBank accession Number | Plant | Function |
| --- | --- | --- |
| AAY18582 | *Medicago truncatula* | isoflavone 7-*O*-methyltransferase |
| AAC49927 | *M. sativa* | isoflavone 7-*O*-methyltransferase |
| BAC58012 | *Glycyrrhiza echinata* | isoflavone 7-*O*-methyltransferase |
| BAC58011 | *G. echinata* | 2,7,4′-trihydroxyisoflavanone 4'-*O*-methyltransferase |
| BAC58013 | *Lotus japonicus* | 2,7,4′-trihydroxyisoflavanone 4'-*O*-methyltransferase |
| AAY18581 | *M.truncatula* | 2,7,4′-trihydroxyisoflavanone 4'-*O*-methyltransferase |
| AAR02420 | *Catharanthus roseus* | flavonoid 4′-*O*-methyltransferase |
| AAR09602 | *Mentha* x *piperita* | flavonoid 4′-*O*-methyltransferase |
| AAR09601 | *Mentha* x *piperita* | flavonoid 3′-*O*-methyltransferase |
| ABB90678 | *Oryza sativa* | flavonoid 3′-*O*-methyltransferase |
| AAA80579 | *Chrysosplenium americanum* | flavonoid 3′-*O*-methyltransferase |
| P59049 | *Chrysosplenium americanum* | Flavonoid/caffeic acid/5-hydroxyferulic acid *O*-methyltransferase |
| AAP23942 | *Triticum aestivum* | flavone *O*-methyltransferase /caffeic acid *O*-methyltransferase |
| ABQ58825 | *Hordeum vulgare subsp. vulgare* | flavonoid 3′, 5′-*O*-methyltransferase |
| ADX43927 | *G. max* | anthocyanin 3′-*O*-methyltransferase |
| AED96460 | *Arabidopsis thaliana* | caffeic acid/5-hydroxyferulic acid *O*-methyltransferase |
| AAF44672 | *Vitis vinifera* | caffeic acid *O*-methyltransferase |
| AES72647 | *M.truncatula* | caffeic acid *O*-methyltransferase |
| AAR09598 | *Mentha* x *piperita* | flavonoid 7-*O*-methyltransferase |
| AAR09600 | *Mentha* x *piperita* | flavonoid 8*-O-*methyltransferase |
